# Natural Genetic Transformation Generates a Population of Merodiploids in *Streptococcus pneumoniae*

**DOI:** 10.1371/journal.pgen.1003819

**Published:** 2013-09-26

**Authors:** Calum Johnston, Stéphanie Caymaris, Aldert Zomer, Hester J. Bootsma, Marc Prudhomme, Chantal Granadel, Peter W. M. Hermans, Patrice Polard, Bernard Martin, Jean-Pierre Claverys

**Affiliations:** 1 Centre National de la Recherche Scientifique, LMGM-UMR5100, Toulouse, France; 2 Université de Toulouse, UPS, Laboratoire de Microbiologie et Génétique Moléculaires, Toulouse, France; 3 Laboratory of Pediatric Infectious Diseases, Radboud University Medical Centre, Nijmegen, The Netherlands; University of Geneva Medical School, Switzerland

## Abstract

Partial duplication of genetic material is prevalent in eukaryotes and provides potential for evolution of new traits. Prokaryotes, which are generally haploid in nature, can evolve new genes by partial chromosome duplication, known as merodiploidy. Little is known about merodiploid formation during genetic exchange processes, although merodiploids have been serendipitously observed in early studies of bacterial transformation. Natural bacterial transformation involves internalization of exogenous donor DNA and its subsequent integration into the recipient genome by homology. It contributes to the remarkable plasticity of the human pathogen *Streptococcus pneumoniae* through intra and interspecies genetic exchange. We report that lethal cassette transformation produced merodiploids possessing both intact and cassette-inactivated copies of the essential target gene, bordered by repeats (R) corresponding to incomplete copies of IS*861*. We show that merodiploidy is transiently stimulated by transformation, and only requires uptake of a ∼3-kb DNA fragment partly repeated in the chromosome. We propose and validate a model for merodiploid formation, providing evidence that tandem-duplication (TD) formation involves unequal crossing-over resulting from alternative pairing and interchromatid integration of R. This unequal crossing-over produces a chromosome dimer, resolution of which generates a chromosome with the TD and an abortive chromosome lacking the duplicated region. We document occurrence of TDs ranging from ∼100 to ∼900 kb in size at various chromosomal locations, including by self-transformation (transformation with recipient chromosomal DNA). We show that self-transformation produces a population containing many different merodiploid cells. Merodiploidy provides opportunities for evolution of new genetic traits via alteration of duplicated genes, unrestricted by functional selective pressure. Transient stimulation of a varied population of merodiploids by transformation, which can be triggered by stresses such as antibiotic treatment in *S. pneumoniae*, reinforces the plasticity potential of this bacterium and transformable species generally.

## Author Summary

Merodiploids are defined as cells possessing a partial duplication of their genetic material, which potentially allows evolution of new genes. Historically, some have been observed in studies of natural genetic transformation. Transformation allows the bacteria to take up foreign DNA and incorporate it into their genome by homology. It is key to the high diversity observed in the human pathogen *Streptococcus pneumoniae* (the pneumococcus). Here we show that transformation with self DNA generates a population of merodiploids with varied chromosomal duplications, up to almost half a genome in size. We show that formation of merodiploids by transformation only requires uptake of a small donor DNA fragment partially repeated in the chromosome. The donor repeat recombines with an alternative repeat on one arm of a replicating chromosome, whilst the non-repeated part recombines with its complement on the other arm, bridging the two. Subsequent recombination events generate a merodiploid chromosome with the region between the two repeats duplicated. Our results demonstrate that transformation, which is induced by stresses such as antibiotic treatments, transiently increases the ability of a population to form merodiploids. We suggest that creating a variety of merodiploids at a time of stress maximizes the adaptive potential of this pathogen.

## Introduction

Partial duplications of genetic material provide potential for evolution of new traits in all kingdoms of life, with duplicated material able to evolve in the absence of functional selective pressure [1]. Such duplications are prevalent in eukaryotes, and were recently shown to be associated with susceptibility to diseases in the human population [2]. Duplications are also thought to provide genetic novelty in plants and yeast [3], [4]. Although diploidy favors gene evolution, prokaryotes are generally haploid in nature, possessing a single copy of most genetic material. Despite this, partial chromosomal duplications, known as merodiploids, allow prokaryotes to evolve new genes. Historically, merodiploidy in bacteria was first suggested in studies of binary encapsulation created by genetic transformation in *Haemophilus influenzae* [5] and *Streptococcus pneumoniae* [6], [7]. Subsequent studies have identified bacterial merodiploids, with various duplications uncovered in *S. pneumoniae* [8], [9], *Bacillus subtilis* [10], *Escherichia coli* [11] and *Salmonella spp.* [12], [13]. However, these studies frequently involved specific markers or chromosomal regions, and the structure and mechanisms of formation of these merodiploids have remained elusive. Extensive analysis conducted in *Salmonella enterica* led to the conclusion that in unselected laboratory cultures 0.005–3% of cells possess duplication of a specific gene [14], with the trade-off between their high formation rate and genetic instability resulting in a steady state after ∼30 generations [15]. However, while it is generally accepted that natural merodiploids occur at relatively high rates in bacteria [16], similar detailed information is not available for any other species.

The most common form of merodiploidy is tandem duplication (TD), where duplicated regions remain adjacent in the chromosome. TDs are generally thought to form spontaneously by homologous recombination between direct repeat sequences such as insertion sequences (IS) or rRNA sequences [17]–[19], an idea supported by the fact that mutation of the recombinase RecA abrogates >90% of TD formation in *S. enterica* [15]. Such homologous recombination events may involve unequal crossing over between different repeat regions of sister chromatids during replication, resulting in production of a TD with a hybrid repeat sequence separating duplicated regions [16]. However, in *S. pneumoniae* the site of spontaneous duplication within the capsule locus appeared to be random and sequences flanking the different duplications were unique [20]. A recent study in *S. enterica* also suggested that RecA-driven recombination may not be the sole mechanism for TD formation. Authors showed that most TDs formed on a replicative plasmid were dependent on an active transposase and conjugative apparatus, and suggested single-strand annealing between transposase-nicked sister chromosomes as an alternative mechanism of TD formation [21].

There is little information regarding TD formation during genetic transfer processes such as genetic transformation, conjugation and transduction. TDs formed by phage transduction have previously been reported but formation was dependent on transduction with non-natural junctions, defined as those not naturally present in the host genome, and therefore somewhat artificial [11], [12], [22]. Similarly, non-natural junctions were used to document formation of duplications during transformation in *B. subtilis* [23]. In the human pathogen *S. pneumoniae*, merodiploids spontaneously generated by transformation have been identified, with cells expressing two polysaccharide capsules isolated [6], [7]. However, the corresponding merodiploid structures have remained undefined and the mechanisms for their formation unknown.

The process of transformation involves internalization of single-stranded (ss) DNA fragments created from an exogenous double-stranded (ds) donor, and their integration into the recipient chromosome by homology. Here, we demonstrate that transformation of *S. pneumoniae* with homologous DNA generates TDs. Formation of these TDs does not require active transposases and is not dependent on non-natural donor junctions but only repeats present in both donor and host DNA. We establish that merodiploid formation involves alternative pairing of partly repeated (R) exogenous sequences resulting in interchromatid integration of internalized R ssDNA. These merodiploids produced by a genetic cross represent true merozygotes and occur throughout the genome. Transformation thus modulates the frequency of merodiploids in a pneumococcal population. These observations reveal a new, non-conservative facet of transformation in the major human pathogen *S. pneumoniae*, reinforcing the view that this species exhibits a great plasticity potential [24]. We discuss both the likelihood that the proposed model, relying on fundamental homologous recombination steps, applies to other transformable species, and the evolutionary potential offered by transformation-generated merodiploidy.

## Results

### A 107.4 kb merodiploid produced by lethal cassette transformation

**Figure 1:**
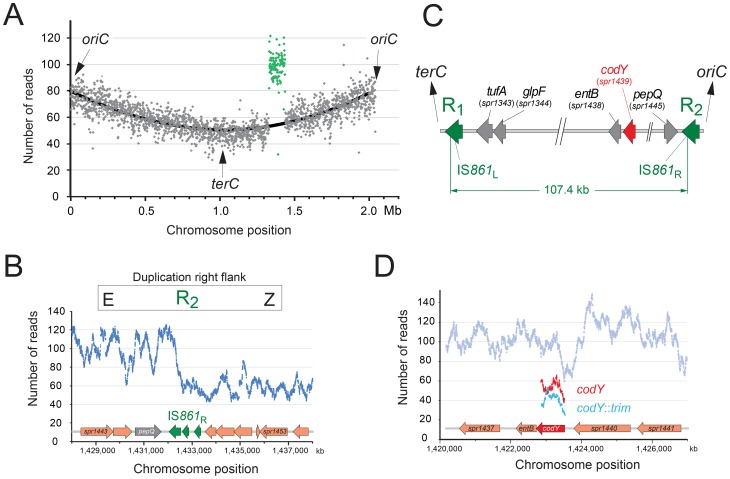
R2597 harbors a duplication of the *codY* chromosomal region. (A) Increased sequence coverage of a 107.4 kb region (green) containing *codY* suggests duplication. (B) Scale map of the duplication limits, bordered by IS*861* sequences. (C) Coverage of the right hand limit of the duplication shows around 1/3 of the IS*861*_R_ is duplicated. (D) Coverage of *codY* and *trim* sequences shows under-representation of *trim*.

**Figure 2:**
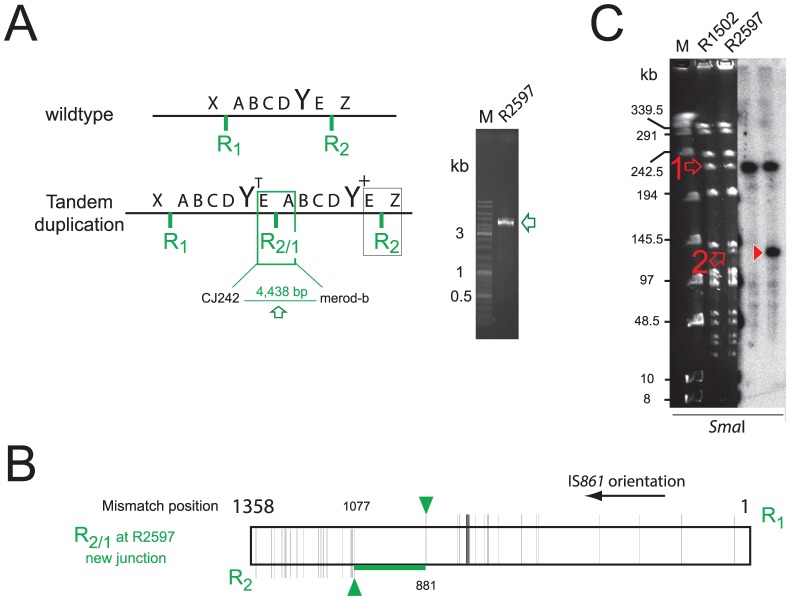
The R2597 duplication is a TD in the chromosome. (A) Predicted R2597 chromosome structure. A–E, duplicated regions; X/Z, flanks; Y, *codY*; R_1_/R_2_ flanking IS*861*; R2/1, predicted hybrid IS*861*. PCR detected the predicted junction in R2597. Green rectangle, TD junction; black rectangle, sequence represented in Figure 1B. (B) Representation of the R_2/1_ hybrid at the TD junction in R2597 identified by sequencing. Mismatches are represented by vertical black lines, extending either above (R_1_) or below (R_2_) the diagram. Horizontal green line represents region within which R_1_ and R_2_ recombined. Green arrows represent limits of recombination between R_1_ and R_2._ (C) PFGE analysis of R2597. Open arrows, restriction fragments predicted in Table S1. Red arrowhead, merodiploid-specific fragment revealed by hybridization. Although the lanes shown were part of the same initial gel/membrane, lanes present between them have been removed to simplify comparison between lanes.

During study of the essential pneumococcal gene *codY*, we identified two independent suppressing mutations allowing survival of otherwise lethal *codY*::*trim* mutant cells in the encapsulated strain D39 [25]. We attempted to recreate this genotype in laboratory strain R1502 by transformation with chromosomal DNA possessing the *codY*::*trim* cassette and the suppressing mutations. In reasonable agreement with our previous report [25], trimethoprim resistant (Trim^R^) transformants appeared with a frequency of ∼0.0020 relative to the *rpsL41* reference marker (conferring resistance to streptomycin, Sm^R^). However, a selected Trim^R^ transformant (R2597) possessed neither suppressing mutation, but harboured both wildtype and *codY*::*trim* loci (Figure S1). This suggested that duplication of the *codY* region allowed R2597 to tolerate inactivation of one copy of *codY*. To identify the extent of the duplication, we sequenced the entire R2597 genome. Results showed that R2597 possesses a large duplication (Figure 1A), bordered by truncated IS*861* sequences (Figure 1B and Figure S2A). This 107.4 kb duplication includes the *codY* region (Figure 1C). Sequence analysis revealed that total coverage of the duplicated region was lower than twice that of a strain lacking the duplication (Figure S2B) and *trim* sequence had lower coverage than *codY* sequence (Figure 1D) indicating that the *codY*::*trim* duplication was under-represented. This suggested instability of the duplicated material, which would be expected from a TD in the chromosome (Figure 2A) or a circular 107.4 kb extrachromosomal element (pop-out; Figure S3AB). Both of these should harbor an identical new junction (E-R_2/1_-A; Figure 2A and Figure S3AB), which was detected by PCR of R2597 (Figure 2A), sequencing of which revealed a chimeric IS*861* made of parts of upstream (R_1_) and downstream (R_2_) IS*861* sequences (Figure 2B).

To distinguish between a chromosomal TD and an extrachromosomal element, we analyzed R2597 by pulse-field gel electrophoresis (PFGE), and hybridization with *codY* and *trim* probes, as different restriction maps were predicted (Table S1). A ∼133-kb *codY*-positive *Sma*I fragment was detected in R2597 (Figure 2C). This band was predicted only for a 107.4 kb TD with a *codY*::*trim*/*codY* ^+^ arrangement; *trim*-specific fragments predicted for this arrangement were also observed (Figure S3C). However, R2597 contained in addition a wildtype-like (224.6-kb) *codY*-positive *Sma*I fragment (Figure 2C). Together with the low band intensity of the ∼133-kb *codY*-positive fragment (Figure 2C), this revealed a mixed population with both non-TD (i.e., wildtype-like) and TD cells present in R2597 culture. Most likely, growth in absence of Trim prior to analyses resulted in partial loss of the duplication, explaining the under-representation observed in both genome sequencing and PFGE. We conclude that an unstable TD of 107.4 kb was created in R2597, rendering it merodiploid for *codY* and allowing mutation of one copy of the gene, and subsequent survival of otherwise lethal recombinants during Trim^R^ selection.

### Self-transformation stimulates merodiploid formation

**Figure 3:**
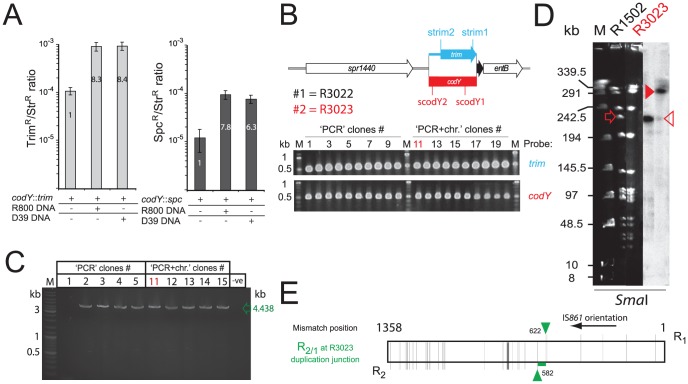
Self-transformation generates merodiploids. (A) Increased survival of lethal transformants after transformation with *codY*::*trim* (grey bars) or *codY*::*spc* (black bars) cassettes in presence of chromosomal DNA from R800 or D39. (B) Maps of *codY+* and *codY*::*trim* loci, and detection of *codY*- (red, scodY1-scodY2 primers) and *trim*-specific (blue, strim1/strim2 primers) sequences by PCR in Trim^R^ clones recovered from transformation with either *codY*::*trim* PCR alone (#1–10), or *codY*::*trim* PCR in presence of R800 chromosomal DNA (#11–20). (C) Detection of TD junction by PCR with CJ242/merod-b primers in clones #1–5 and #11–15 from Figure 3B. 9/10 of these clones possessed the TD junction. The one clone without the TD junction (clone #1, R3022) was found to possess an additive insertion of the *codY*::*trim* cassette in close proximity to the *codY* locus, creating a very small TD (See Figure S4B–E). (D) PFGE analysis of chromosomal DNA from clone #11 (R3023), digested by *Sma*I and hybridized with *codY*-specific probe. Open red arrow, expected wild-type *codY* ethidium bromide stained band; closed red arrowhead, R3023 *codY* fragment, expected for *codY* ^+^/*codY*::*trim* merodiploid; open red arrowhead, wild-type *codY* band weakly present in R3023. Although the lanes shown were part of the same initial gel/membrane, lanes present between them have been removed to simplify comparison between lanes. (E) Representation of the R_2/1_ hybrid at the TD junction in R3023 identified by sequencing, layout as in Figure 2B.

R2597 was isolated following transformation with chromosomal DNA (containing *codY*::*trim*). To determine whether a sequence in the chromosomal DNA could stimulate merodiploid formation, we transformed cells with *codY*::*trim* (conferring trimethoprim resistance) or *codY*::*spc* (conferring spectinomycin resistance) PCR fragments in the presence or absence of isogenic chromosomal DNA. Co-transformation of chromosomal DNA stimulated formation of merodiploids between 6 and 8-fold (Figure 3A). PCR analysis of 10 clones recovered from the *codY*::*trim* transformation or the *codY*::*trim* plus R800 DNA transformation showed that both *codY* and *trim* sequences were present in all clones (Figure 3B). Out of 10 clones tested (5 from each transformation), 9 possessed the TD junction (Figure 3C). PFGE and hybridization analysis, and sequencing of the junction of clone 11 (R3023) confirmed the presence of a 107.4 kb TD but with a *codY* ^+^/*codY*::*trim* arrangement instead of the opposite arrangement observed with R2597 (Figure 3DE, Figure S4A and S4C, and Table S1).

Analysis of clone 1 (R3022), lacking the TD junction, uncovered a small duplication of the *codY* locus with a *codY* ^+^/*codY*::*trim* arrangement lacking flanking repeats (duplication of 3,276 bp, 1,422,105–1,425,381 on R6 genome, Figure S4B–E). Although the mechanisms of formation of such a duplication remain unclear, similar small duplications lacking flanking repeats have previously been documented in *S. pneumoniae* [20]. We confirmed formation of merodiploids independently of competence in pneumococcal cells by detecting the TD junction by PCR in a strain unable to develop competence (R1501; unpublished observations). These results suggested firstly that merodiploids occur at basal levels in a *S. pneumoniae* population, accounting for the appearance of transformants with only *codY*::*trim* or *codY*::*spc* PCR fragments as donor, and secondly that transformation transiently stimulated their formation. However, the alternative possibility that transformation was facilitating, by some ill-defined mechanism, the emergence of pre-existing merodiploids could not be formally excluded.

### Transformation with R-NRf fragments triggers merodiploidy

**Figure 4:**
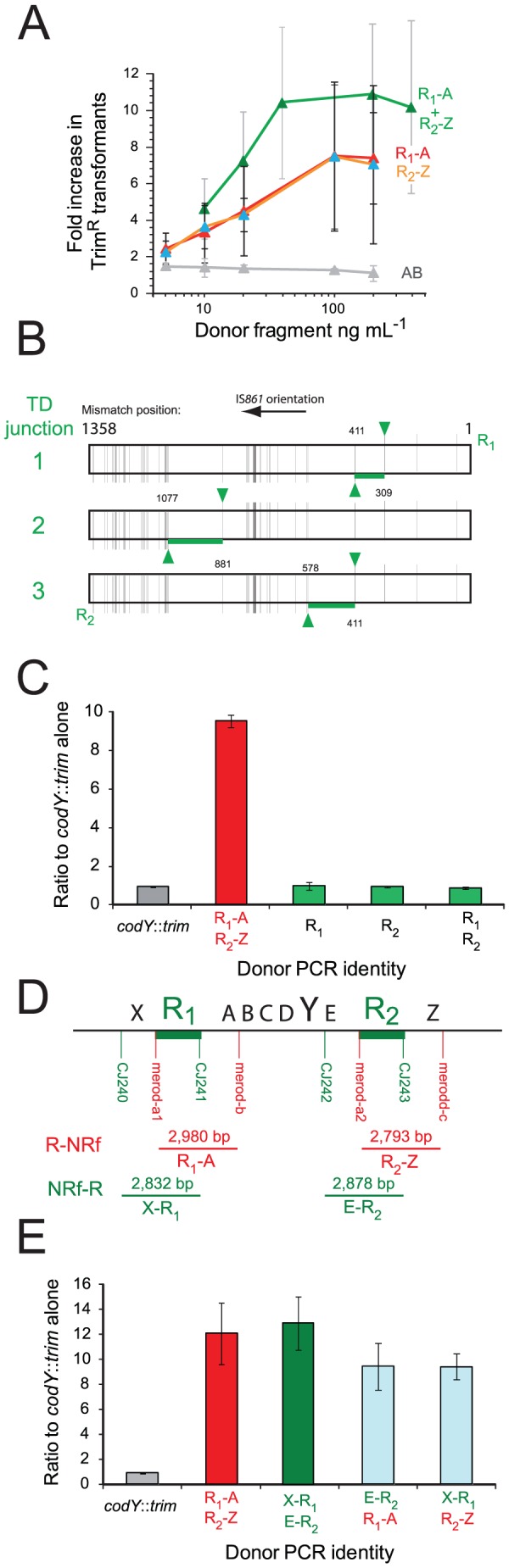
R-NRf donor fragments promote merodiploid formation, allowing survival of otherwise lethal *codY*::*trim* transformants. (A) Co-transformation of 100 ng mL^−1^ *codY*::*trim* with R_1_-A (red), R_2_-Z (orange), R_1_-A and R_2_-Z (green) or comparable non-R-NRf fragments (AB, grey). (B) R TD junction sequence of three independent clones. Layout as in Figure 2B. (C) Donor fragments with only repeats do not induce merodiploidy. DNA concentrations: 100 ng mL^−1^ for *codY*::*trim* cassette with 100 ng mL^−1^ of R1 or R2 PCR fragment; 50 ng mL^−1^ R1-A and 50 ng mL^−1^ R2-Z as positive control. PCR primers used: R1, merod-a1/CJ241; R2, merod-a2/CJ243. (D) Diagrammatic representation of the *codY* chromosomal region with location of primers used to produce PCR fragments assayed in transformation in combination with the *codY*::*trim* cassette (panel E). (E) Cooperativity requires two R-NRf fragments with same polarity. DNA concentrations: 100 ng mL^−1^ for *codY*::*trim* cassette; 50 ng mL^−1^ of each R-NRf fragment in each test, giving a total of 100 ng mL^−1^ fragment per test.

Transformation-induced merodiploidy was investigated further with the aim of distinguishing between the two possibilities, duplication made by transformation or simple trapping of a pre-existing duplication. Since the identified TDs were flanked by *IS*861 repeat sequences (Figure 2A), and separated by a hybrid *IS*861 sequence (Figure 2B), we hypothesized that an alternative pairing event between donor DNA and host chromosome, followed by an unequal crossing over, could generate merodiploids during transformation. This would be dependent on a donor fragment containing R as well as associated non-repeated flanking sequence (NR-f). To test this, we amplified R-NRf PCR fragments representing R_1_-A and R_2_-Z (Figure 2A) and co-transformed cells with *codY*::*trim* and R_1_-A or R_2_-Z. Co-transformation increased the frequency of *codY*::*trim* ‘lethal cassette’ transformants up to 8-fold (Figure 4A) compared to transformation with *codY*::*trim* alone. All tested clones possessed the same TD, and sequencing of the junction of three of them identified three distinct R_2/1_ recombination junctions (Figure 4B). Identical transformation with similarly-sized PCR fragments lacking repeat sequences, AB, had no effect on frequency of lethal cassette transformants (Figure 4A). Furthermore, donor fragments containing R_1_ or R_2_ alone (i.e., without NRf) also had no effect (Figure 4C), showing that the R-NRf ‘hybrid’ structure of the donor fragment is key to merodiploid formation.

### Model for formation of merodiploids by transformation

**Figure 5:**
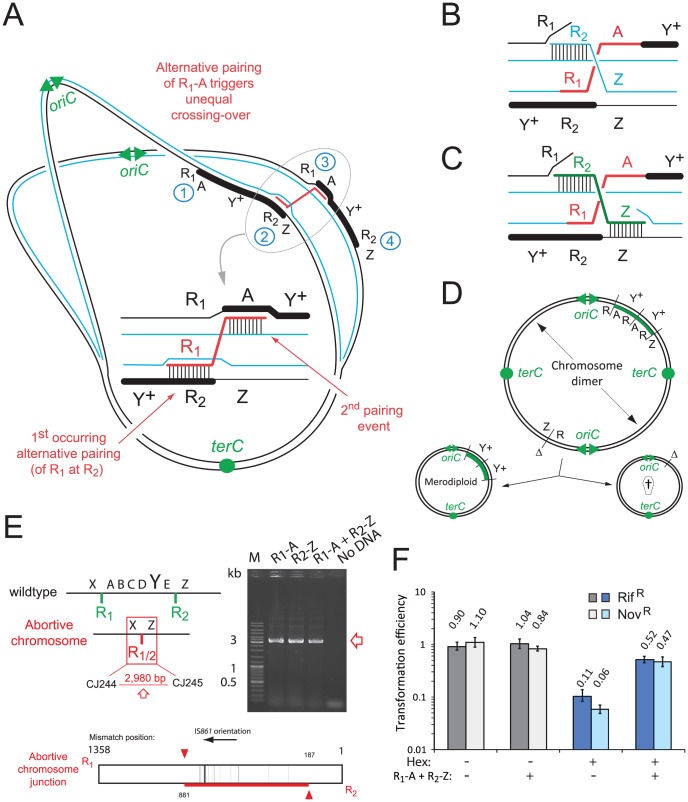
Model of merodiploid formation during pneumococcal transformation. (A) Alternative pairing between R-NRf sequences triggers unequal crossing over. Letters as in Figure 1D. (B) Spontaneous restoration of complementary strand integrity. (C) Restoration of complementary strand integrity stimulated by R_2_-Z donor fragment. (D) Resolution of chromosome dimer to produce one tandem-duplicated chromosome and one abortive chromosome. *oriC*, origin of replication; *terC*, terminus of replication; Δ, deletion; †, abortive chromosome. (E) Predicted abortive chromosome structure. Layout as in Figure 2A. Red square; abortive chromosome junction. Detection of abortive chromosome junction by primers in Figure 3C. Diagram of R_1/2_ sequence at abortive chromosome junction amplified after R_1_-A transformation, determined by sequencing of PCR fragments. Vertical lines, R_1_/R_2_ mismatches; red horizontal line, mixed sequence .(F) Saturation of the Hex system establishes frequent alternative pairing of R-NRf fragment. Transformation efficiencies of Rif^R^ and Nov^R^ point mutations (carried by R304 chromosomal DNA) compared in *hex^−^* (R246) or *hex^+^* (R800) recipients in the presence or absence of R-NRf PCR fragments. Transformation efficiencies normalized to Sm^R^ (*rpsL41*) point mutation, resistant to Hex. *hex* ^−^ recipient, shades of gray; *hex* ^+^ recipient, shades of blue.

Based on these observations, we elaborated a general model for merodiploid formation by transformation (Figure 5A), where alternative pairing of R-NRf donor fragments (red) leads to interchromatid integration, bridging the neosynthesized (blue) and parental (black) chromatids of a partially replicated chromosome. Alternative pairing is crucial to our model, occurring here by initial pairing of donor R_1_ with R_2_ in the host chromosome, displacing the neosynthesized strand, followed by pairing of flanking A with A on the sister chromatid (Figure 5A). Subsequent restoration of sister chromatid continuity (Figure 5B) results in unequal crossing-over, creating a chromosome dimer. Resolution of this dimer generates a merodiploid chromosome (possessing two copies of *codY*) and an abortive chromosome lacking *codY* (Figure 5D). Our results showed that R_1_-A and R_2_-Z were equally efficient at generating merodiploids (Figure 4A). We suggest that this is because R_1_-A can bridge chromatids associating loci 2 and 3 and R_2_-Z can bridge loci 1 and 4 (Figure 5A), the resulting recombinant chromosomes being indistinguishable.

The break in the complementary chromatid resulting from interchromatid integration of R_1_-A can be repaired spontaneously through invasion of the sister chromatid by the displaced R_2_ neosynthesized strand (blue) (Figure 5B). We also envisioned an alternative donor DNA-directed break repair mechanism involving interchromatid integration of donor R_2_-Z (Figure 5C). R_2_-Z fragment integration would require displacement of both the parental R_1_ strand (creating R_1/2_) and neosynthesized Z strand, bridging the gap between chromatids. Consistent with this model, transformation with both R_1_-A and R_2_-Z donor fragments increased the frequency of Trim^R^ transformants by up to 10-fold, an increased efficiency compared to either fragment alone (Figure 4A). To determine whether observed cooperativity of donor fragments was dependent on polarity, we co-transformed cells with combinations of four donor fragments (R_1_-A, R_2_-Z, X-R_1_, E-R_2_; Figures 2A and 4D). Results showed that only donor fragments of the same polarity are able to cooperate to restore complementary strand integrity during merodiploid formation (Figure 4E). Thus, restoration of complementary strand integrity can occur either spontaneously or via interchromatid integration of an exogenous fragment, allowing chromosome dimer creation, and subsequent merodiploid formation (Figure 5D).

A predicted consequence of the repair of the complementary chromatid is the formation of an abortive chromosome, subsequent to the creation of the X-R_1/2_-Z junction (Figure 5E). We were able to detect this abortive chromosome junction by specific PCR on unselected cultures 30 minutes after transformation with R-NRf fragments (Figure 5E), showing that this junction is indeed produced. Analysis of the junction sequence produced by PCR on R_1_-A-transformed culture showed a long region of mixed sequence (Figure 5E), suggesting that a number of different alternative pairing events had occurred within the population of merodiploids produced.

### Alternative pairing of R fragments is frequent

While sequencing of junctions provided evidence for alternative pairing of R segments (Figures 2B, 3E and 4B), the overall frequency of such events in the transformed population remained unknown. To further validate our model and obtain an estimate of this frequency, we took advantage of a previous observation that the generalized mismatch repair system (Hex) of *S. pneumoniae* can be saturated by excess mismatches [26], [27]. Hex operates on transformation intermediates, ejecting donor DNA from transformation heteroduplexes when 1–2 mismatches are present [28], [29]. A larger number of mismatches can result in saturation of Hex, rendering it unable to repair a single mismatch elsewhere in the genome. The R_1_ and R_2_ fragments share 96% sequence identity, with the 4% divergence a target for Hex if alternative pairing occurs. We therefore investigated the ability of these fragments to saturate Hex by comparing transformation efficiency of two Hex sensitive point mutations, *rif23* and *nov1* (conferring resistance to rifampicin, Rif^R^, and to novobiocin, Nov^R^, respectively) on chromosomal donor DNA with or without R-NRf fragments. Addition of R-NRf fragments resulted in a net increase in Rif^R^ and Nov^R^ transformation efficiencies in a *hex^+^* recipient, indicative of escape from mismatch repair (Figure 5F). We conclude that Hex was saturated in >50% of cells, establishing that alternative pairing is a frequent event when a culture is transformed with a DNA fragment harboring a region repeated in the recipient chromosome.

### Transformation does not select pre-existing structures but creates *de novo* merodiploids

**Figure 6:**
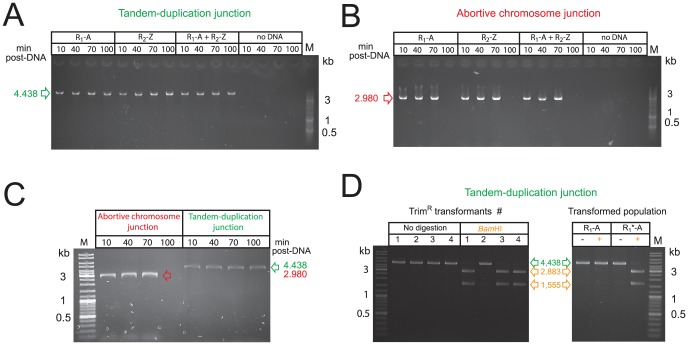
Transformation does not select pre-existing structures but creates *de novo* merodiploids. (A) Detection of tandem-duplication junction by PCR on cultures at different time points after transformation with R-NRf fragments alone. (B) Detection of abortive chromosome junction on same transformed cultures as in panel A. (C) Detection of tandem-duplication and abortive chromosome junctions by PCR on cultures at different time points after self-transformation. (D) Integration of donor R_1_*-A fragment during merodiploid formation. Right panel shows *Bam*HI restriction of TD junction PCRs recovered from Trim^R^ clones transformed with *codY*::*trim* and R_1_*-A PCR fragments. Undigested PCR shown in green, products of *Bam*HI digestion shown in orange. Left panel shows *Bam*HI digestion of TD junction PCR amplified from population transformed with R_1_-A or R_1_*-A. −, undigested PCR fragment; +, *Bam*HI-digested PCR fragment.

Detection of abortive chromosome junctions on unselected cells shortly after transformation (Figure 5E) supported the idea of merodiploid formation by transformation irrespective of lethal selection. To provide further evidence for this, we detected TD and abortive chromosome junctions by PCR on transformed cultures at different times post-transformation with R-NRf fragments. The TD junction was readily detected in the transformed population as early as 10 min after DNA internalization (Figure 6A). Such timing is consistent with previous data indicating completion of ssDNA integration within ∼10 min after uptake [30]. This result confirmed that R-NRf fragments promote merodiploidy in the absence of the *codY*::*trim* lethal cassette and therefore independently of selection. Since self-transformation generated the same merodiploids as R-NRf transformation (Figure 3), TD junctions were expected to be present in cultures transformed with self DNA in the absence of selection. PCR on self-transformed cultures confirmed their presence (Figure 6C) supporting the view that transformation creates *de novo* merodiploids.

The abortive chromosome was detected by PCR on the same transformed cultures (Figure 6B). Similarly to TD junctions, abortive chromosome junctions were readily detected in cultures transformed with self DNA in the absence of selection (Figure 6C). Interestingly, the abortive chromosome junctions disappeared 100 minutes after uptake, presumably due to cell division, and loss of abortive cells, whereas TD junctions persisted (Figure 6B and 6C).

To definitely prove that the detected junctions were created by transformation and specifically depend upon physical integration of the exogenous R-NRf donor fragment, we mutated three bases at the 5′ end of R in R_1_-A to insert a restriction site (*Bam*HI), creating R_1_*-A. We transformed cells with *codY*::*trim* and R_1_*-A PCR fragments, and recovered Trim^R^ clones. TD junction PCRs of 3/4 tested clones were sensitive to *Bam*HI restriction, confirming integration of donor R_1_*-A (Figure 6D, left panel). The single insensitive PCR fragment was presumably derived from a spontaneous TD. Furthermore, PCR fragments amplified from cultures transformed with R_1_*-A alone were sensitive to *Bam*HI restriction, confirming integration of the R_1_*-A donor fragment in the absence of any selection (Figure 6D, right panel). Taken together, these results demonstrate that transformation does not simply select pre-existing merodiploids from a population but actively promotes formation of *de novo* merodiploids.

### Transformation creates merodiploids throughout the genome

**Figure 7:**
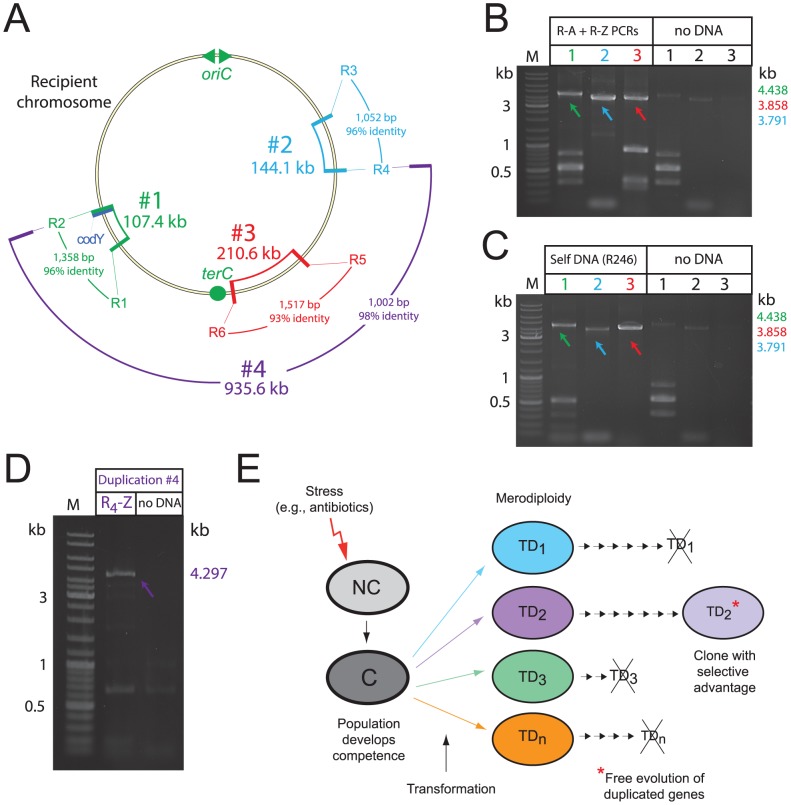
Transformation-triggered merodiploidy is a general process. (A) Chromosomal location of generated TDs. Size, identity and homology of repeat sequences noted. (B) PCR detection of TD junctions in the transformed population 40 min after uptake of appropriate R-NRf fragments (R_1_-A+R_2_-Z; R_3_-A+R_4_-Z; R_5_-A+R_6_-Z). (C) PCR detection of TD junctions in the transformed population 40 min after self-transformation. PCRs carried out on same culture of transformed cells. Control PCRs without transforming DNA on the same cells as in Figure 5B. (D) Detection of TD #4 junction by PCR with primers in Figure S5G, PCR carried out on population transformed with R_4_-Z fragment. (E) Schematic representation of proposed evolutionary potential of merodiploidy triggered by transformation. Stresses such as antibiotics can induce competence in *S. pneumoniae* and other species [48]. NC, non-competent; C, competent; TD, tandem-duplication where numbers represent different merodiploids. Small arrows indicate subsequent cell divisions. Crossed-out TD indicates loss of duplication due to intrinsic instability.

In order to determine whether creation of merodiploids by transformation is a general phenomenon, we investigated the triggering of merodiploidy at different chromosomal sites. We identified two other pairs of truncated IS repeat regions, each sharing >90% homology and separated respectively by 144.1 (site #2) and 210.6 (site #3) kb (Figure 7A and Figure S5AB). Transformation with R-NRf fragments corresponding to sites #2 and #3 triggered appropriate merodiploid formation, detected by PCR specific for the TD junctions (i.e., E-R_4/3_-A and E-R_6/5_-A; Figure S5CD) (Figure 7B). Sequencing of these junctions identified R_4/3_ and R_6/5_ mixed recombination sites (Figure S5EF).

Self-transformation should also provide the recipient population with the R-NRf fragments required to produce any of the TDs shown in Figure 7A. PCR detection of junctions specific for site #1-3 TDs on the same self-transformed cells harvested 40 minutes after uptake of isogenic chromosomal DNA confirmed that all three TDs were created within the same transformed population (Figure 7C). We conclude that self-transformation is capable of simultaneously producing a large variety of merodiploids in a population.

Finally, to determine whether formation of a much larger merodiploid could be stimulated by transformation, we transformed cells with the R_4_-Z donor fragment, and selected Trim^R^ transformants. R_4_ shares 98% identity with R_2_ over 1002 bp, and alternative pairing of donor R_4_ with chromosomal R_2_ should promote formation of a 935.6 kb merodiploid, duplicating *codY* and allowing insertion of *codY*::*trim* (Figure 7A). R_4_-Z increased transformation efficiency of *codY*::*trim* PCR 3-fold, suggesting merodiploid formation (data not shown). This was confirmed by transformation with only R_4_-Z and detection of the specific TD junction (E-R_2/4_-A; Figure S5G) by PCR on transformed culture (Figure 7D). Sequencing of this TD junction confirmed R_2/4_ recombination (Figure S5H). These results demonstrate that transformation-induced merodiploidy is a general process which can occur at many chromosomal locations, and that over 40% of the pneumococcal genome can be duplicated as the result of the uptake and processing of a single short R-NRf ssDNA fragment.

## Discussion

Starting from a case study, the characterization of a merodiploid transformant obtained by mutating the essential pneumococcal gene *codY* [25], we provide evidence that merodiploidy promoted by self-transformation is a general phenomenon in the pneumococcus. Merodiploid formation relies on transformation with a donor DNA fragment consisting of R, a segment repeated in the recipient chromosome, flanked by NRf, its non-repeated flank. The mechanistic model proposed and validated in this study relies on alternative pairing of R (i.e., pairing at a secondary chromosomal copy) allowing subsequent homologous NRf pairing on the sister chromatid, which leads to interchromatid integration of the R-NRf exogenous fragment (Figure 5A–C). The resulting unequal crossing-over simultaneously creates a TD and generates a chromosome dimer, which upon resolution produces a chromosome with the TD and an abortive chromosome lacking the duplicated region (Figure 5D). Supporting this model, transformation with R-NRf fragments promoted predictable merodiploid formation (Figure 4A), with TD and abortive chromosome junctions identified (Figures 4B, 5E and 6). Furthermore, evidence for frequent alternative pairing of R was obtained (Figure 5F) and specific chromosomal integration of donor R was confirmed (Figure 6D).

### Underlying mechanisms of merodiploid formation by transformation

Alternative pairing of R is a crucial step of our model (Figure 5A). RecA-directed homology search being random, R presumably has an equal chance of pairing with each chromosomal repeat and our data show that alternative pairing is frequent, occurring in >50% of cells (Figure 5F). Whilst pairing initiating at the ‘normal’ site (defined by its NRf) would result in RecA-driven synapsis extending into the NRf region, alternative pairing of R occurring first allows NRf to pair with its homologue in the sister chromatid, the second key feature of our model (Figure 5A). Such pairing is presumably facilitated by three-dimensional homology search [31] involving intersegmental contact sampling catalyzed by RecA [32]. Alternative pairing leading to interchromatid integration introduces a break in the complementary chromatid (Figure 5B). Restoration of complementary strand integrity, required to recover a double-stranded chromosome structure, can occur through alternative pairing and interchromatid integration of a second independently taken up R-NRf fragment (Figures 5C and 4A).

Restoration of complementary strand integrity can also occur spontaneously (Figure 5B). Owing to the conservation in nature of annealing of ssDNA displaced by recombinase-mediated strand exchange [33], annealing of the displaced R recipient strand to the sister chromatid R single strand by RecO could readily take place. Alternatively, this annealing could be catalyzed by the transformation-dedicated RecA loader, DprA, which has this ability [34]. Restoration of strand continuity produces a chromosome dimer which, upon resolution, generates both a merodiploid and an abortive chromosome (Figure 5D). Dimer resolution prior to division of the transformant could involve either site-specific recombination [35] or homologous recombination. In any case, merodiploidy triggered by transformation is a complex process associating both ssDNA integration (as a trigger) and subsequent dsDNA processing mechanisms. Formed by recombination during a genetic exchange process, these merodiploids are thus true merozygotes, defined as bacterial cells containing a second copy of part of the chromosome.

### A single short donor fragment triggers >900 kb-long duplications

Remarkably, alternative pairing of a donor fragment as short as ∼3 kb triggers duplication of long chromosomal regions, ranging from the 107.4 kb TD observed in the *codY*::*trim* merodiploid R2597 (Figures 1 and 2) up to 935.6 kb (Figure 7A). This is reminiscent of an old observation that P1 phage transduction allowed the production of a transductant in which a duplication much larger than the quantity of P1 phage genetic material was produced [11]. The authors concluded that “…the requirements for transduction of the “condition of merodiploidy” appear to be the cotransduction of the (TD junction)…” and implicated “A mechanism whereby two recipient chromosomes interact with the transduced (junction)… to regenerate the TD …”. This outcome is in essence very similar to what we describe, although the mechanisms involved differ significantly. Merodiploid formation by transformation, involving internalization and processing of ssDNA rather than dsDNA, does not require uptake of a pre-existing TD junction but only a small R-NRf fragment. Interchromatid integration of this fragment is sufficient to trigger a cascade of recombination/repair events, ultimately leading to production of a merodiploid chromosome with a long TD. This process potentially occurs at many places in the pneumococcal chromosome, as demonstrated in Figure 7.

### Efficiency of merodiploid formation by transformation

The frequency of duplications in a growing bacterial population is considered to range in *Escherichia coli* and *Salmonella typhimurium* from >10^−2^ to ∼10^−4^ [16], [17], and 0.005–3% of cells in an unselected laboratory culture of *Salmonella enterica* were estimated to contain duplication of a specified gene [14]. In *S. pneumoniae*, transformation frequencies with *codY*::*trim* PCR alone (Figure 3A) suggest that ∼0.05% of the recipient cells already possessed a *codY* duplication. We showed that 4 out of 5 transformants tested had the same TD junction as the transformation-generated merodiploids (Figure 3C). *S. pneumoniae* thus has basal levels of merodiploidy in noncompetent cells, and transformation can be viewed as a mechanism that transiently augments the formation of merodiploids in a population. For merodiploids generated by transformation with a R-NRf fragment, a rough estimate of the expected frequency of *codY*::*trim* transformants, assuming that every alternative pairing event promoted merodiploid formation (i.e., that each cell could accept the lethal cassette), indicates that the observed frequency is between 100 and 1000-fold lower than this expectation. This could result from a failure to restore complementary strand integrity and/or a reduced probability of interchromatid interactions, hence of interchromatid pairing of NRf, due to nucleoid organization and/or choreography [36]. Nevertheless, the isolation of the R2597 merodiploid after transformation with chromosomal DNA carrying the lethal *codY*::*trim* cassette and two independent suppressors, rather than a transformant having acquired the two suppressors, indicates that merodiploid formation is more frequent than simultaneous transformation by two independent mutations.

### Merodiploidy and mutation of essential genes

The initial merodiploid in this study was identified via transformation with chromosomal DNA containing a lethal cassette, mutating the essential *codY* gene [25]. We have shown that both pre-existing and transformation-generated merodiploids allowed tolerance of the otherwise lethal *codY*::*trim* cassette. These observations show that the study of essential pneumococcal genes through inactivation by transformation carries its own potential drawback, as merodiploidy can obscure the results. This was illustrated when the *clpX* gene, encoding part of the Clp protease in *S. pneumoniae*, was shown to be an essential gene [37] after initial publication of a *clpX* mutant strain [38]. Authors suggested that mutation of *clpX* occurred in a merodiploid cell which maintained wild-type and mutated copies of the gene [37]. Coupled with our observations that a basal level of spontaneous merodiploid formation exists within a population and that transformation with self DNA can transiently increase the formation of merodiploids, these results suggest that great care should be taken when attempting to mutate genes with important roles in pneumococcal physiology, and that rigorous validation of successful mutation is essential.

### Evolutionary potential of transformation-triggered merodiploidy

We have demonstrated that pneumococcal transformation increases the formation of merodiploids. It is thus particularly relevant that competence for genetic transformation is induced in response to antibiotics [39], constituting a pneumococcal SOS substitute [30]. This provides the pneumococcus with maximum potential for plasticity, including merodiploid formation, at a time when adaptation is crucial for survival. Interestingly, the increase in merodiploidy via transformation does not require the presence of non-self exogenous DNA but readily occurs with chromosomal DNA that could be released by daughter cells in the culture. Thus, the occurrence of fratricide within pneumococcal cultures [40] could provide the competent cells encountering adverse conditions with a transiently increased adaptive potential subsequent to transformation-generated gene duplication. The production of merodiploids by self-transformation thus constitutes a new, non-conservative facet of pneumococcal transformation in the sense that it creates novel junctions (the TD junction). In addition, the resulting gene duplications, allowing mutation of duplicated material without the constraints of selective pressure, are likely to be of great evolution potential [16]. We conclude that merodiploidy stimulated by transformation produces a wide variety of merodiploids within a population, maximising the adaptive potential of the transformed population in response to conditions of stress (Figure 7E).

### Merodiploidy triggered by self-transformation, a mechanism shared by all transformable species?

Our validated model for merodiploid formation relies on canonical homologous recombination and repair steps. These are likely to be shared by any organism. This is supported by the fact that the vast majority of duplications (>90%) are RecA-dependent [15] and none of the steps leading to merodiploidy during pneumococcal transformation differ from those documented in *E. coli* or *Salmonella* species [11], [14], [17]. In light of the conservation of the transformation machinery, including the presence of the transformation-dedicated RecA loader DprA in transformable species [34], [41], it is likely that merodiploidy can be triggered by transformation in most species. The previous findings that chromosomal integration of foreign DNA linked, on one side, to a piece of DNA homologous to the recipient chromosome occurred with similar characteristics in species as phylogenetically distant as *S. pneumoniae*, *Acinetobacter* sp. and *Pseudomonas stutzeri* [42]–[44] further support the view that basic transformation mechanisms are conserved. It is therefore quite likely that merodiploidy promoted by self-transformation is a transient plasticity mechanism shared by all transformable species.

## Materials and Methods

### Bacterial strains, culture and transformation conditions

*S. pneumoniae* strains and primers are described in Table S2. CSP-induced transformation was performed as described previously [45], using precompetent cells treated at 37°C for 10 min with synthetic CSP1 (100 ng mL^−1^). After addition of transforming DNA, cells were incubated for 20 min at 30°C. Transformants were selected by plating on CAT-agar supplemented with 4% horse blood, followed by challenge with a 10 mL overlay containing kanamycin (250 µg mL^−1^), Nov (4 µg mL^−1^), Rif (2 µg mL^−1^), spectinomycin (100 µg mL^−1^), Sm (200 µg mL^−1^) or Trim (20 µg mL^−1^), after phenotypic expression for 120 min at 37°C.

Specifics of experiments for merodiploid formation with R-NRf PCR fragments, for creation and detection of IS*861* TD and AC junctions, for detection of other TD junctions and for self-transformation are described in Text S1.

### Whole-genome sequencing of D39 and D39Δ*codY*

Roche 454 FLX whole genome sequencing was performed by LGC Genomics (Berlin, Germany) using genomic DNA isolated from mid-log cultures by the Genomic DNA kit (Qiagen). For each strain, a single read library and a 3-kb span paired-end library were generated and sequenced according to Roche standard protocols. A total of 451,127 reads (96,405 of which contained paired ends passing quality filtering) were obtained for R1502 (59-fold coverage), and 367,697 reads with 137,475 paired ends were obtained for R2597 (64-fold coverage).

Data from the sequencing runs were mapped to the reference R6 strain (Acc.no.: NC_003098) using Roche GsMapper [Release 2.3 (091027_1459)], and coverage data was extracted from the alignment results. Sequence coverage was defined as the number of times any given genomic base is represented in sequence reads. The loess function as implemented in the Loess R package was used to plot a smoothed line of the coverage as a function of genomic position.

### PFGE and hybridization

PFGE analysis was based on a published protocol [46], [47]. Strains were grown in THY medium until OD_550_ 0.2, and digestions carried out with *Apa*I, *Sac*II and *Sma*I enzymes. To create hybridization probes, *codY* or *trim* PCR fragments were amplified with scodY1/scodY2 (D39) or strim1/strim2 (TD80) primer pairs.

## Supporting Information

Figure S1 Further analysis of the R2597 merodiploid strain. (A) Structure of the *codY^+^* and *codY*::*trim* loci. Primers used for *codY^+^* (red color) and *codY*::*trim* (blue color) region in panel B are indicated, together with predicted fragment sizes. *codY*::*trim* PCR fragments are 6 bp shorter than *codY* ^+^ fragments. *codY* ^+^ (codYatg and codY4) and *trim* (trim1 and trim2) specific primers were used to generate PCR fragments discriminating between *codY* ^+^ and *codY*::*trim*. Relevant restriction sites introduced with the *trim* cassette are also indicated. (B) PCR probing of the structure of the *codY* region in strains TD80 (*codY*::*trim* donor) and R2597 (Trim^R^ transformant). Lanes C and D, *trim*-specific fragments (blue arrowheads); lanes A and E, *codY^+^*-specific fragments (red arrowheads). M, kb ladder.
(EPS)

Figure S2 Further analysis of whole genome sequence of R2597. (A) Coverage of the left hand limit of the duplication shows around 2/3 of the IS*861*_L_ is duplicated. (B) Estimation of the coverage of the extra copy of the *codY* chromosomal region. The coverage for the duplicated region was estimated by using locally weighted scatterplot smoothing (LOESS) on coverage as a function of genomic position. Coverage of the duplicated material (green dots) is lower than for the *terC* region, suggesting under-representation of the duplicated region at the time total DNA was extracted for genome sequencing.
(PDF)

Figure S3 Further analysis of R2597 by PFGE, and predicted restriction maps. (A) Predicted restriction map of *codY^+^* extrachromosomal element. (B) Predicted restriction map of *codY*::*trim* extrachromosomal element. (**C**) PFGE analysis of R2597. Chromosomal DNA of strains R1502 and R2597 was digested with *Sma*I, *Sac*II and *Apa*I restriction enzymes and probed with *codY* or a mixture of *codY* and *trim* (1∶1 ratio) probes. Both ethidium-bromide-stained gel and corresponding hybridization are shown. M, kb ladder (*Hin*dIII-digested λ DNA). Open arrows, restriction fragments, predicted in Table S1. Arrowheads, fragments revealed by hybridization; blue color, *trim* specific; red color, *codY* specific supernumerary fragment. *Sma*I PFGE with *codY* hybridization can be found in Figure 1E. Although the lanes shown were part of the same initial gel/membrane, lanes present between them have been removed to simplify comparison between lanes. (**D**) Predicted restriction map of wild-type chromosome covering duplicated region. (**E**) Predicted restriction map of R2597, containing a *codY*::*trim*/*codY^+^* TD.
(EPS)

Figure S4 Further analysis of R3022 and R3023 by PFGE, and predicted restriction maps. (A) Predicted restriction map of R3023, containing a *codY^+^*/*codY*::*trim* TD. (B) Restriction map of R3022, containing a very small *codY^+^*/*codY*::*trim* TD. (C) PFGE and hybridization analysis of clones R3022 and R3023, as for R2597 in Figure S3C. Hybridization of chromosomal DNA with *codY*-specific probe and digestion by *Sma*I, *Apa*I and *Sac*II. Open red arrows, wildtype ethidium bromide-stained bands; closed red arrowheads, *codY^+^*/*codY*::*trim*-specific bands and hybridizations in R3023; open red arrowheads; faint wildtype bands in R3023 sample; closed red arrowheads, *codY*-specific bands in R3022. All band sizes as predicted in Table S1. Although the lanes shown were part of the same initial gel/membrane, lanes present between them have been removed to simplify comparison between lanes. (D) Scale map of TD region in R3022 strain. Primers codY6 and trim2 used to amplify 3,277 bp TD-specific fragment. (E). PCR amplification of small TD-specific junction from R3022, absent from wild-type and R3023 merodiploid controls.
(EPS)

Figure S5 Identities of TDs #2, #3 and #4. (A) Scale map of TD #2 duplication limits. (B) Scale map of TD #3 duplication limits. (C) Predicted chromosome structure of TD #2. A–E, duplicated regions; R_3_/R_4_, IS*861* repeats; blue rectangle, TD junction, with primers used to amplify, and fragment size indicated. (D) Predicted chromosome structure of TD #3. A–E, duplicated regions; R_5_/R_6_, IS*1167* repeats; red rectangle, TD junction, with primers used to amplify, and fragment size indicated. (E) Hybrid R_4/3_ sequence of TD junction #2. Layout as in Figure 2B. (F) Hybrid R_6/5_ sequence of TD junction #3. Layout as in Figure 2B. (G) Predicted chromosome structure of TD #4. A–E, duplicated regions; R_2_/R_4_, IS*861* repeats; violet rectangle, TD junction, with primers used to amplify, and fragment size indicated. (H) Hybrid R_2/4_ sequence of TD junction #4. Layout as in Figure 2B.
(EPS)

Table S1 Predicted restriction maps of the *codY* region(s) with or without he 107.4 kb duplication*.
(DOCX)

Table S2 Strains and primers used in this study.
(DOCX)

Text S1 Supplementary Materials and Methods.
(DOCX)

## References

[1] Bergthorsson U, Andersson DI, Roth JR (2007) Ohno's dilemma: evolution of new genes under continuous selection. Proc Natl Acad Sci U S A 104: 17004–17009. 17942681 10.1073/pnas.0707158104 PMC2040452

[2] Conrad DF, Pinto D, Redon R, Feuk L, Gokcumen O, et al (2010) Origins and functional impact of copy number variation in the human genome. Nature 464: 704–712. 19812545 10.1038/nature08516 PMC3330748

[3] Flagel LE, Wendel JF (2009) Gene duplication and evolutionary novelty in plants. New Phytol 183: 557–564. 19555435 10.1111/j.1469-8137.2009.02923.x

[4] Hittinger CT, Carroll SB (2007) Gene duplication and the adaptive evolution of a classic genetic switch. Nature 449: 677–681. 17928853 10.1038/nature06151

[5] Leidy G, Hahn E, Alexander HE (1953) In vitro production of new types of *Hemophilus influenzae* . J Exp Med 97: 467–482. 13052813 10.1084/jem.97.4.467 PMC2136293

[6] Austrian R, Bernheimer HP (1959) Simultaneous production of two capsular polysaccharides by pneumococcus. I. Properties of a pneumococcus manifesting binary capsulation. J Exp Med 110: 571–584. 13795198 10.1084/jem.110.4.571 PMC2137005

[7] Bernheimer HP, Wermundsen IE (1969) Unstable binary capsulated transformants in pneumococcus. J Bacteriol 98: 1073–1079. 4389232 10.1128/jb.98.3.1073-1079.1969 PMC315298

[8] Kashmiri SV, Hotchkiss RD (1975) Evidence of tandem duplication of genes in a merodiploid region of Pneumococcal mutants resistant to sulfonamide. Genetics 81: 21–31. 1323 10.1093/genetics/81.1.21 PMC1213385

[9] Ravin AW, Takahashi EA (1970) Merodiploid ribosomal loci arising by transformation and mutation in pneumococcus. J Bacteriol 101: 38–52. 4391557 10.1128/jb.101.1.38-52.1970 PMC250448

[10] Audit C, Anagnostopoulos C (1973) Genetic studies relating to the production of transformed clones diploid in the tryptophan region of the *Bacillus subtilis* genome. J Bacteriol 114: 18–27. 4633342 10.1128/jb.114.1.18-27.1973 PMC251735

[11] Hill CW, Schiffer D, Berg P (1969) Transduction of merodiploidy: induced duplication of recipient genes. J Bacteriol 99: 274–278. 4895847 10.1128/jb.99.1.274-278.1969 PMC249999

[12] Anderson RP, Miller CG, Roth JR (1976) Tandem duplications of the histidine operon observed following generalized transduction in *Salmonella typhimurium* . J Mol Biol 105: 201–218. 787532 10.1016/0022-2836(76)90107-8

[13] Lehner AF, Hill CW (1980) Involvement of ribosomal ribonucleic acid operons in *Salmonella typhimurium* chromosomal rearrangements. J Bacteriol 143: 492–498. 6156935 10.1128/jb.143.1.492-498.1980 PMC294275

[14] Anderson RP, Roth JR (1977) Tandem genetic duplications in phage and bacteria. Annu Rev Microbiol 31: 473–505. 334045 10.1146/annurev.mi.31.100177.002353

[15] Reams AB, Kofoid E, Savageau M, Roth JR (2010) Duplication frequency in a population of *Salmonella enterica* rapidly approaches steady state with or without recombination. Genetics 184: 1077–1094. 20083614 10.1534/genetics.109.111963 PMC2865909

[16] Andersson DI, Hughes D (2009) Gene amplification and adaptive evolution in bacteria. Annu Rev Genet 43: 167–195. 19686082 10.1146/annurev-genet-102108-134805

[17] Anderson P, Roth J (1981) Spontaneous tandem genetic duplications in *Salmonella typhimurium* arise by unequal recombination between rRNA (rrn) cistrons. Proc Natl Acad Sci U S A 78: 3113–3117. 6789329 10.1073/pnas.78.5.3113 PMC319510

[18] Haack KR, Roth JR (1995) Recombination between chromosomal IS200 elements supports frequent duplication formation in *Salmonella typhimurium* . Genetics 141: 1245–1252. 8601470 10.1093/genetics/141.4.1245 PMC1206863

[19] Flores M, Mavingui P, Perret X, Broughton WJ, Romero D, et al (2000) Prediction, identification, and artificial selection of DNA rearrangements in *Rhizobium*: toward a natural genomic design. Proc Natl Acad Sci U S A 97: 9138–9143. 10922070 10.1073/pnas.97.16.9138 PMC16835

[20] Waite RD, Struthers JK, Dowson CG (2001) Spontaneous sequence duplication within an open reading frame of the pneumococcal type 3 capsule locus causes high-frequency phase variation. Mol Microbiol 42: 1223–1232. 11886554 10.1046/j.1365-2958.2001.02674.x

[21] Reams AB, Kofoid E, Kugelberg E, Roth JR (2012) Multiple pathways of duplication formation with and without recombination (RecA) in *Salmonella enterica* . Genetics 192: 397–415. 22865732 10.1534/genetics.112.142570 PMC3454872

[22] Hughes KT, Roth JR (1985) Directed formation of deletions and duplications using Mud(Ap, lac). Genetics 109: 263–282. 3156064 10.1093/genetics/109.2.263 PMC1202487

[23] Niaudet B, Janniere L, Ehrlich SD (1985) Integration of linear, heterologous DNA molecules into the *Bacillus subtilis* chromosome: mechanism and use in induction of predictable rearrangements. J Bacteriol 163: 111–120. 3924889 10.1128/jb.163.1.111-120.1985 PMC219087

[24] Claverys JP, Prudhomme M, Mortier-Barrière I, Martin B (2000) Adaptation to the environment: *Streptococcus pneumoniae*, a paradigm for recombination-mediated genetic plasticity? Mol Microbiol 35: 251–259. 10652087 10.1046/j.1365-2958.2000.01718.x

[25] Caymaris S, Bootsma HJ, Martin B, Hermans PWM, Prudhomme M, et al (2010) The global nutritional regulator CodY is an essential protein in the human pathogen *Streptococcus pneumoniae* . Mol Microbiol 78: 344–360. 20979332 10.1111/j.1365-2958.2010.07339.x

[26] Guild WR, Shoemaker NB (1974) Intracellular competition for a mismatch recognition system and marker specific rescue of transforming DNA from inactivation by ultraviolet irradiation. Mol Gen Genet 128: 291–300. 4150369 10.1007/BF00268517

[27] Humbert O, Prudhomme M, Hakenbeck R, Dowson CG, Claverys JP (1995) Homeologous recombination and mismatch repair during transformation in *Streptococcus pneumoniae*: saturation of the Hex mismatch repair system. Proc Natl Acad Sci USA 92: 9052–9056. 7568071 10.1073/pnas.92.20.9052 PMC40922

[28] Claverys JP, Lacks SA (1986) Heteroduplex deoxyribonucleic acid base mismatch repair in bacteria. Microbiol Rev 50: 133–165. 3523187 10.1128/mr.50.2.133-165.1986 PMC373061

[29] Claverys JP, Méjean V, Gasc AM, Sicard AM (1983) Mismatch repair in *Streptococcus pneumoniae*: relationship between base mismatches and transformation efficiencies. Proc Natl Acad Sci USA 80: 5956–5960. 6310606 10.1073/pnas.80.19.5956 PMC390196

[30] Claverys JP, Martin B, Polard P (2009) The genetic transformation machinery: composition, localization and mechanism. FEMS Microbiol Rev 33: 643–656. 19228200 10.1111/j.1574-6976.2009.00164.x

[31] Gonda DK, Radding CM (1986) The mechanism of the search for homology promoted by recA protein. Facilitated diffusion within nucleoprotein networks. J Biol Chem 261: 13087–13096. 3020024

[32] Forget AL, Kowalczykowski SC (2012) Single-molecule imaging of DNA pairing by RecA reveals a three-dimensional homology search. Nature 482: 423–427. 22318518 10.1038/nature10782 PMC3288143

[33] Sugiyama T, Kantake N, Wu Y, Kowalczykowski SC (2006) Rad52-mediated DNA annealing after Rad51-mediated DNA strand exchange promotes second ssDNA capture. EMBO J 25: 5539–5548. 17093500 10.1038/sj.emboj.7601412 PMC1679760

[34] Mortier-Barrière I, Velten M, Dupaigne P, Mirouze N, Piétrement O, et al (2007) A key presynaptic role in transformation for a widespread bacterial protein: DprA conveys incoming ssDNA to RecA. Cell 130: 824–836. 17803906 10.1016/j.cell.2007.07.038

[35] Le Bourgeois P, Bugarel M, Campo N, veran-Mingot ML, Labonte J, et al (2007) The unconventional Xer recombination machinery of Streptococci/Lactococci. PLoS Genet 3: e117. 17630835 10.1371/journal.pgen.0030117 PMC1914069

[36] Reyes-Lamothe R, Nicolas E, Sherratt DJ (2012) Chromosome replication and segregation in bacteria. Annu Rev Genet 46: 121–143. 22934648 10.1146/annurev-genet-110711-155421

[37] Robertson GT, Ng WL, Gilmour R, Winkler ME (2003) Essentiality of *clpX*, but not *clpP*, *clpL*, *clpC*, or *clpE*, in *Streptococcus pneumoniae* R6. J Bacteriol 185: 2961–2966. 12700276 10.1128/JB.185.9.2961-2966.2003 PMC154392

[38] Robertson GT, Ng WL, Foley J, Gilmour R, Winkler ME (2002) Global transcriptional analysis of *clpP* mutations of type 2 *Streptococcus pneumoniae* and their effects on physiology and virulence. J Bacteriol 184: 3508–3520. 12057945 10.1128/JB.184.13.3508-3520.2002 PMC135132

[39] Prudhomme M, Attaiech L, Sanchez G, Martin B, Claverys JP (2006) Antibiotic stress induces genetic transformability in the human pathogen *Streptococcus pneumoniae* . Science 313: 89–92. 16825569 10.1126/science.1127912

[40] Claverys JP, Håvarstein LS (2007) Cannibalism and fratricide: mechanisms and raisons d'être. Nat Rev Microbiol 5: 219–229. 17277796 10.1038/nrmicro1613

[41] Quevillon-Cheruel S, Campo N, Mirouze N, Mortier-Barrière I, Brooks MA, et al (2012) Structure-function analysis of pneumococcal DprA protein reveals that dimerization is crucial for loading RecA recombinase onto DNA during transformation. Proc Natl Acad Sci USA 109: E2466–E2475. 22904190 10.1073/pnas.1205638109 PMC3443122

[42] Prudhomme M, Libante V, Claverys JP (2002) Homologous recombination at the border: insertion-deletions and the trapping of foreign DNA in *Streptococcus pneumoniae* . Proc Natl Acad Sci U S A 99: 2100–2105. 11854505 10.1073/pnas.032262999 PMC122325

[43] De Vries J, Wackernagel W (2002) Integration of foreign DNA during natural transformation of *Acinetobacter* sp. by homology-facilitated illegitimate recombination. Proc Natl Acad Sci U S A 99: 2094–2099. 11854504 10.1073/pnas.042263399 PMC122324

[44] Meier P, Wackernagel W (2003) Mechanisms of homology-facilitated illegitimate recombination for foreign DNA acquisition in transformable *Pseudomonas stutzeri* . Mol Microbiol 48: 1107–1118. 12753199 10.1046/j.1365-2958.2003.03498.x

[45] Martin B, Prudhomme M, Alloing G, Granadel C, Claverys JP (2000) Cross-regulation of competence pheromone production and export in the early control of transformation in *Streptococcus pneumoniae* . Mol Microbiol 38: 867–878. 11115120 10.1046/j.1365-2958.2000.02187.x

[46] Le Bourgeois P, Mata M, Ritzenthaler P (1989) Genome comparison of *Lactococcus* strains by pulsed-field gel electrophoresis. FEMS Microbiol Lett 59: 65–69. 2737464 10.1016/0378-1097(89)90460-6

[47] Le Bourgeois P, Lautier M, van den BL, Gasson MJ, Ritzenthaler P (1995) Physical and genetic map of the *Lactococcus lactis* subsp. cremoris MG1363 chromosome: comparison with that of *Lactococcus lactis* subsp. *lactis* IL 1403 reveals a large genome inversion. J Bacteriol 177: 2840–2850. 7751295 10.1128/jb.177.10.2840-2850.1995 PMC176957

[48] Charpentier X, Polard P, Claverys JP (2012) Induction of competence for genetic transformation by antibiotics: convergent evolution of stress responses in distant bacterial species lacking SOS? Curr Op Microbiol 15: 1–7. 10.1016/j.mib.2012.08.001 22910199

