# Supplementary figures and images for: Natural Genetic Transformation Generates a Population of Merodiploids in Streptococcus pneumoniae
Source: PLoS Genet. 2013 Sep 26;9(9):e1003819. doi: 10.1371/journal.pgen.1003819 (PMC3784515; doi:10.1371/journal.pgen.1003819)

A

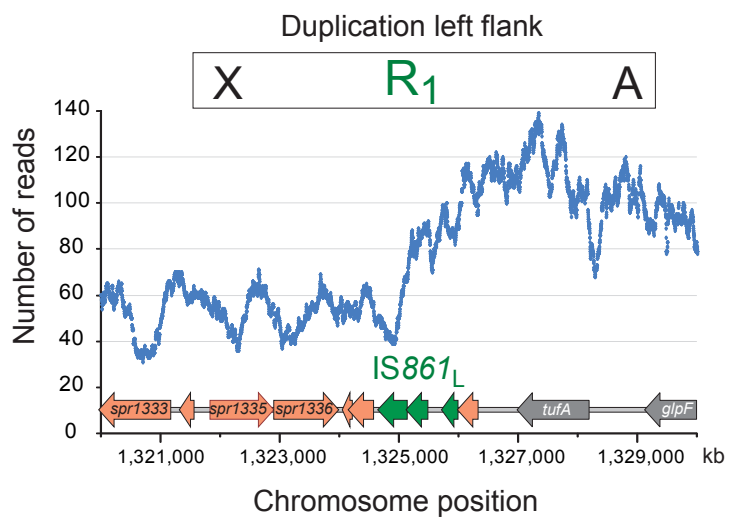

B

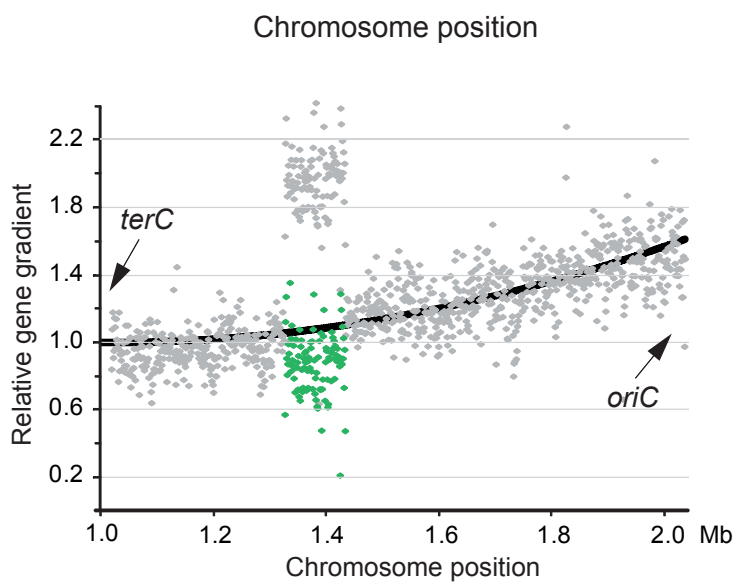

Supplement: Figure S2 — Further analysis of whole genome sequence of R2597. (A) Coverage of the left hand limit of the duplication shows around 2/3 of the IS861 L is duplicated. (B) Estimation of the coverage of the extra copy of the codY chromosomal region. The coverage for the duplicated region was estimated by using locally weighted scatterplot smoothing (LOESS) on coverage as a function of genomic position. Coverage of the duplicated material (green dots) is lower than for the terC region, suggesting under-representation of the duplicated region at the time total DNA was extracted for genome sequencing. (PDF) [file pgen.1003819.s002.pdf]
