# Supplementary material for: Natural Genetic Transformation Generates a Population of Merodiploids in Streptococcus pneumoniae
Source: PLoS Genet. 2013 Sep 26;9(9):e1003819. doi: 10.1371/journal.pgen.1003819 (PMC3784515; doi:10.1371/journal.pgen.1003819)
Supplement: Table S1 — Predicted restriction maps of the codY region(s) with or without he 107.4 kb duplication*. (DOCX) [file pgen.1003819.s006.docx]

| **Line** | **Prototype**  **genome** | **Extra material** | **Duplication arrangement** | **Size (kb) of restriction fragments hybridizing with** | | | | | |
| --- | --- | --- | --- | --- | --- | --- | --- | --- | --- |
|  |  |  |  | ***codY*^+^ probe**^a^ | | | ***codY*::*trim* probe** | | |
|  |  |  |  | ***Sma*I** | ***Apa*I** | ***Sac*II** | ***Sma*I** | ***Apa*I** | ***Sac*II** |
| **#1** | **R6 (R1502)** | **none** | na^b^ | 224,593 | 44,627 | 97,504 | - | - | - |
| **#2** | **R2597**^c^ | **TD** | *codY*::*trim* / *codY*^+^ | 133,120^d^ | 44,627 | 97,504 | 198,861 | 95,289^d^ | 78,966 |
| **#3** | **R3023** | **TD** | *codY*^+^ /  *codY*::*trim* | 306,255^d^ | 104,830^d^ | 107,394^d^ | 306,255^d^ | 35,086 | 78,966 |
| **#4** | **na** | *codY*^+^  **pop-out circle** | na | no site^e^ | 104,830^d^ | 107,394^d^ | - | - | - |
| **#5** | **na** | *codY*::*trim* **pop-out circle** | na | - | - | - | 107,388^d^ | 95,289^d^ | 78,966 |
| **#6** | **R3022** | **TD (no flanking repeats)** | *codY*^+^ /  *codY*::*trim* | 202,138 | 12,812 | 82,243 | 202,138 | 35,086 | 82,243 |

* Maps with absolute coordinates of *Sma*I, *Apa*I and *Sac*II restriction sites in the published R6 genome can be found in Supplementary Figure 3A (*codY*^+^ pop-out), Supplementary Figure 3B (*codY*::*trim* pop-out), Supplementary Figure 3D (R6), Supplementary Figure 3E (R2597), Supplementary Figure 4A (R3023) and Supplementary Figure 4C (R3022) respectively.

^a^ Fragments differing from those present in the wildtype parent (line #1) are indicated in red colour

^b^ not applicable

^c^PFGE analysis revealed the presence of a mixed population of wildtype-like cells and cells harboring the indicated TD in R2597 culture, i.e., fragments predicted in lines #1 and #2 (Figure 2C and Supplementary Figure 3C)

^d^ Fragment sizes calculated based on the TD junction identified in the R2597 clone. In the case of a different junction, such as the junction in R3023, where more of the R_2_ repeat is present, the fragment sizes will decrease by 10 bp

^e^ We assume such a large molecule would be present in the form of open-circle and/or 107.4 kb linear fragment depending on the extent of spontaneous nicking
