# Supplementary material for: Natural Genetic Transformation Generates a Population of Merodiploids in Streptococcus pneumoniae
Source: PLoS Genet. 2013 Sep 26;9(9):e1003819. doi: 10.1371/journal.pgen.1003819 (PMC3784515; doi:10.1371/journal.pgen.1003819)
Supplement: Table S2 — Strains and primers used in this study. (DOCX) [file pgen.1003819.s007.docx]

|  |  |  |
| --- | --- | --- |
|  |  |  |
| ***S. pneumoniae*  strain** | **Genotype/Description** | **Source Reference** |
|  |  |  |
| D39 | Serotype 2 | NCTC 7466 |
| D39∆*cps*∆*codY* | D39 but ∆*cps*::*kan*, ∆*codY*::*trim*, *socY*^a^; Kan^R^, Trim^R^, Mtx^R^ | [49] |
| R246 | R800 but *hexA*Δ*3*::*ermAM*; Ery^R^ | [34] |
| R304 | R800 derivative, *nov1, rif23, rpsL41*; NovR, Rif^R^, Sm^R^ | [34] |
| R800 | R6 derivative | [50] |
| R1501 | R800 but ∆*comC* | [51] |
| R1502 | R800 ∆*comC, ssbB*::*luc* (*ssbB*^+^); Cm^R^ | [51] |
| R2597^¶^ | R1502 but ∆*codY*::*trim, codY*^+^; Cm^R^, Trim^R^ | This study |
| R3022^¶^ | R1501 but ∆*codY*::*trim*, *codY*^+^; Trim^R^ | This study |
| R3023^§^ | R1501 but ∆*codY*::*trim*, *codY*^+^; Trim^R^ | This study |
| TD80 | D39*∆cps∆codY* but *rpsL41*; Kan^R^, Sm^R^, Trim^R^, Mtx^R^ | [25] |
| TD81 | TD80 but *codY*::*spc3*^A^; Kan^R^, Spc^R^, Sm^R^, Mtx^R^ | [25] |
|  |  |  |
| **Primers** | **Sequence^‡^; (position^p^)** |  |
|  |  |  |
| CJ240 | CGGAACTTGCTGGACAGATTTTTGA; (1,323,145-1,323,169) | This study |
| CJ241 | GTCTCAATTCAAGAAGACAAGTGAAC; (1,326,002-1,325,977) | This study |
| CJ242 | CAGTGACGTCAAAAGCAAGGC; (1,430,484-1,430,504) | This study |
| CJ243 | TAATATAGGATGTTGAAATGATGA; (1,433,385-1,433,362) | This study |
| CJ244 | TAGCCTAGAATGTGTCGTAAACTT; (1,323,806-1,323,829) | This study |
| CJ245 | ATGATGGACAGACCTTTACTATCCT; (1,434,193-1,434,169) | This study |
| CJ250 | AACGATCGTTGCCACGACTTCCTTTTTTG; (1,423,889-1,423,917) | This study |
| CJ251 | AAATACACTCGGAGCTAAGTTAGTAG; (1,422,215-1,422,190) | This study |
| CJ252 | CATAGTGTTTCTCTGTGAATGCCTGTT; (352,588-352,614) | This study |
| CJ253 | TGCTATTGGAGAAGCTCCTGTTAATG; (355,184-355,159) | This study |
| CJ254 | CATAGTATTTCTCTGTAAAGGCCTGTT; (496,667-496,703) | This study |
| CJ255 | CCTGTCCCGCATGAAGTAAACCATT; (499,226-499,202) | This study |
| CJ256 | TCTATGCAACCAACTTACAACATTGA; (495,457-495,482) | This study |
| CJ257 | GAAGAAAAGCTAAGCTCGAGAAAGG; (761,016-761,040) | This study |
| CJ258 | AACGAGTCATGCTCTTACCATTGTCA; (763,932-763,907) | This study |
| CJ259 | TCTCAGTAAAGAAGCTAAAAAATCCC; (971,166-971,191) | This study |
| CJ260 | GTAAGATGCACGAAGACTGTTAATT; (974,080-974,056) | This study |
| CJ261 | CCATCTGTAGGGAAGTCAACACTTT; (970,200-970,224) | This study |
| CJ276 | AAAGTTAGATTTTTTCTGTCTAACTTTTGGGGATCCGTTCACTTGTCTTC; (1,325,941-1,325,990) | This study |
| CJ277 | GAAGACAAGTGAACGGATCCCCAAAAGTTAGACAGAAAAAATCTAACTTT; (1,325,990-1,325,941) | This study |
| codY1 | CAAGGATCAGTTTTCCCATATTTTCG; (1,421,888-1,421,913) | [25] |
| codY2 | CTTCGTGTCCTTCGTGACTTTA; (1,424,528-1,424,507) | [25] |
| codY4 | CAGAGGAGCAGTTGCAGGATG; (1,423,472-1,423,452) | This study |
| codY6 | GTTAAGAAGCTCAATCCCAACAAC; (1,423,045-1,423,068) | This study |
| codY7 | ATTTCACCAGTCAATGCTTTCACA; (1,419,506-1,419,529) | This study |
| codY8 | AAATGAAAACGCTTTCTAGGCAAA; (1,426,900-1,426,877) | This study |
| codY10s | ATAGACCGCATGGTTTACCC; (1,417,659-1,417,678) | This study |
| codY10as | AATGCCGACAGTGAGGGCAG; (1,428,598-1,428,579) | This study |
| codYatg | TGAATCATGACACATTTATTAGAAAAAACTAG; (1,423,520-1,423,499) | [25] |
| codYdo | CAAATAGAAAAATTGCCAAACCAGA; (1,417,468-1,417,492) | This study |
| codYrbs | ATCCTCATGATTAAATTCGAGGTGAAAAATGGC; (1,423,541-1,423,520) | [25] |
| codYup | TCTTTATCAAATTGAAGGGCGACT; (1,428,507-1,428,484) | This study |
| codYstop | AAATTGGATCCTTTGTCATTAGTAATCTCTTTTC; (1,422,729-1,422,751) | This study |
| glyQ1 | GAGTGCTCATACTCAGGCTGGATAA; (1,316,946-1,316,970) | This study |
| grtB1 | GTCCAATCGTTTTTCTAAGGGACTC; (1,440,204-1,440,180) | This study |
| merod-a1 | CTACCGAAACCAGCCTCATCTTG; (1,324,548-1,324-570) | This study |
| merod-a2 | GTATCAGGTGTTGAATGTACTGCCC; (1,432,002-1,432,026) | This study |
| merod-b | CCAGAACCAGAACGTGACACTGAC; (1,327,551-1,327,528) | This study |
| merod-c | GAAGGACAGGCAACTTGCAGGTC; (1,434,817-1,434,795) | This study |
| MP188 | TTCATTTTCACCAACCAGGTTAC; (1,422,142-1,422,164) | [25] |
| MP189 | ATTGGCTGCTGAGTTTACTCCAG; (1,424,142-1,424,120) | [25] |
| pgdA1 | GGATGTGGATAGTCTGGACTGGAAG; (1,319,967-1,319,943) | This study |
| scodY1 | GGTGACAGCAGTACGACGAC; (1,422,994-1,423,013) | This study |
| scodY2 | TTACATCAATTTTGAAACGC; (1,423,474-1,423,493) | This study |
| strim1 | CCGGTACCGTTACGACGCGC; *(499-480)* | This study |
| strim2 | ATGAACCCGGAATCGGTCCG; *(1-19)* | This study |
| trim1 | GGTTTGGTAAGGGCTTGCCTAT; *(186-165)* | This study |
| trim2 | GGAGCCGAGGTATATGCGCT; *(309-328)* | This study |
| 1454R | GGATGACTTTCTCTTGATTTGCTTG; (1,437,198-1,437,222) | This study |
|  |  |  |
|  |  |  |
| ^a^*socY* (suppressor of *codY*) indicates the presence of *fatC^C496T^* and *amiC^G1459T^* mutations allowing survival of *codY* mutant cells {25] | | |
| ^R^, Resistance; Cm, chloramphenicol; Ery, erythromycin; Kan, kanamycin, Mtx, methotrexate; Spc, spectinomycin | | |
| ^¶^Strain with merodiploid *codY*^+/trim^ chromosome | |  |
| ^§^Strain with merodiploid *codY^trim/+^* chromosome | |  |
| ^C^ and ^A^ indicate, respectively, the co-transcribed and reverse orientation of an inserted mini-transposon antibiotic resistance | | |
| cassette with respect to the target gene | |  |
| ^‡^Underlined bases indicate restriction sites used for cloning in this study | |  |
| ^p^Oligonucleotide position given on the R6 genome sequence [52], or in reference to the *trim* orf start (italics) | | |

49. Hendriksen WT, Bootsma HJ, Estevão S, Hoogenboezem T, De Jong A et al. (2008) CodY of *Streptococcus pneumoniae*: link between nutritional gene regulation and virulence. J Bacteriol 190: 590-601.

50. Lefèvre J-C, Mostachfi P, Gasc A-M, Guillot E, Pasta F et al. (1989) Conversion of deletions during recombination in pneumococcal transformation. Genetics 123: 455-464.

51. Dagkessamanskaia A, Moscoso M, Hénard V, Guiral S, Overweg K et al. (2004) Interconnection of competence, stress and CiaR regulons in *Streptococcus pneumoniae*: competence triggers stationary phase autolysis of *ciaR* mutant cells. Mol Microbiol 51: 1071-1086.

52. Hoskins J, Alborn WE, Jr., Arnold J, Blaszczak LC, Burgett S et al. (2001) Genome of the bacterium *Streptococcus pneumoniae* strain R6. J Bacteriol 183: 5709-5717.
