# Supplementary material for: Natural Genetic Transformation Generates a Population of Merodiploids in Streptococcus pneumoniae
Source: PLoS Genet. 2013 Sep 26;9(9):e1003819. doi: 10.1371/journal.pgen.1003819 (PMC3784515; doi:10.1371/journal.pgen.1003819)
Supplement: Text S1 — Supplementary Materials and Methods. (DOCX) [file pgen.1003819.s008.docx]

Supplementary Materials and Methods

**Merodiploid Formation by Transformation with R-RNf PCR Fragments**

Merodiploid formation was investigated by transformation of R246 with 100 ng mL^-1^ *codY*::*trim* PCR (codY7/codY8 primers, 7,371 bp) in the presence of different R-NRf PCR fragments (total IS*861* PCR concentration 100 ng mL^-1^, 50:50 split if two PCRs used). Three different PCR primer pairs were used to amplify IS*861*_L_ with its 5’ flank (X-R_1_, CJ240/CJ241 primers, 2,832 bp), 3’ flank (R_1_-A, merod-a1/merod-b primers, 2,980 bp), or no flank (R_1_, merod-a1/CJ241 primers, 1,429 bp). Similarly, three different PCR primer pairs were used to amplify IS*861*_R_ with its 5’ flank (E-R_2_, CJ242/CJ243 primers, 2,878 bp), 3’ flank (R_2_-Z, merod-a2/merod-c primers, 2,793 bp), or no flank (R_2_, merod-a2/CJ243 primers, 1,360 bp). Fragments of DNA of similar size in the vicinity of these IS*861* repeats were amplified (upstream, glyQ1/pgdA1 primers, 2,997 bp; downstream, 1454R/grtB1 primers, 2,982 bp) and used as control donor PCRs.

**Creation and Detection of IS*861* TD and Abortive Chromosome Junctions**

To create and detect the 107.4 kb TD, R246 cultures were transformed in the classical manner described above, with 100 ng mL^-1^ of *codY*::*trim* PCR (codY7/codY8 primers, 7,371 bp) and 100 ng mL^-1^ of R_1_-A PCR, or R_1_-A and R_2_-Z PCRs together. Individual Trim^R^ transformants were grown in C+Y + 20 µg mL^-1^ Trim, and used as template for a diagnostic PCR (CJ242/merod-b primers), amplifying a 4,438 bp junction only if the TD is present. Primers CJ241 and merod-a2 were used for sequencing.

To detect the transient presence of the abortive chromosome (107.4 kb deletion, *codY^-^*), as well as the TD in the same transformants, 1 mL R246 cultures were transformed with either no DNA, 100 ng mL^-1^ of R_1_-A PCR, 100 ng mL^-1^ of R_2_-Z PCR, a mixture of 50 ng mL^-1^ of each PCR (R_1_-A + R_2_-Z). Cells were left to grow in liquid medium for phenotypic expression. 200 µL volumes of culture were removed 10, 40, 70 and 100 min after DNA uptake, pelleted, and resuspended in 10 µL C+Y medium (+ 15% glycerol). Diagnostic PCRs were then carried out on 2 µL of these cells (CJ244/CJ245 primers), amplifying a 2,980 bp fragment of DNA specific to the abortive chromosome junction. Primers CJ243 and merod-a1 were used for sequencing. The TD junction was detected by PCR on the same cells.

**Detection of Other TD Junctions** Appropriate R-A and R-Z fragments were amplified, with around 1,200-1,500 bp of R and 1,500 bp of NRf (IS*861* R_3_-A = CJ252/CJ253 352,588-353,614 in R6 genome, R_4_-Z = CJ254/CJ255 496,677-497,692; IS1167 R_5_-A = CJ257/CJ258 761,074-762,401, R_6_-Z = CJ259/CJ260 971,225-972,527). R246 competent cells were transformed as above with 100 ng mL^-1^ of these PCR fragments, before liquid phenotypic expression. 200 uL of culture were harvested 40 min after DNA uptake, pelleted, and resuspended in 10 µL C+Y medium (+ 15% glycerol). Specific diagnostic PCRs were then carried out on 2 µL of these cells (IS*861*, CJ253/CJ256, 3,971 bp; IS*1167*, CJ258/CJ261, 3,858 bp). TD junction PCRs to detect the 935.6 kb merodiploid were carried out with primers CJ242/CJ255, 4,408 bp).

**Self-transformation**

R246 competent cells were transformed as above with 2 µL of R246 chromosomal DNA (‘self’), followed by liquid phenotypic expression. 200 µL volumes of culture were removed 10, 40, 70 and 100 min after DNA uptake, pelleted, and resuspended in 10 µL C+Y medium (+ 15% glycerol). Diagnostic PCRs for the *codY*-IS*861* TD (CJ242/merod-b, 4,438 bp) and abortive chromosome (CJ244/CJ245, 2,980 bp) junctions were carried out on 2 µL of these cells. To confirm general merodiploid formation in these cells, PCRs to detect region #2 IS*861* (CJ253/CJ256, 3,971 bp) and region #3 IS*1167* (CJ258/CJ261, 3,858 bp) TD junctions were carried out.

**Splicing overlap extension (SOE) PCR**

To create the artificial R-NRf fragment R_1_*-A, R_1_ and A were individually amplified by PCR with primers overlapping by 50 bp and introducing a *Bam*HI site (GGATCC) at the 3’ end of R_1_, replacing bases GGTGCA at positions 1,325,971-1,325,976 in the R6 genome (R_1_: merod-a1/CJ277, 1,443 bp; A: merod-b/CJ276, 1,611 bp). These PCR products were then used as template in tandem in a PCR to create a 3,054 hybrid DNA fragment representing R_1_*-A and consisting of R1-A with a *Bam*HI siteat the 3’ end of R_1_. This fragment was used in transformation studies to generate merodiploids, and specific TD junction PCRs were confirmed to possess the *Bam*HI restriction site, confirming integration of R_1_*-A.
